# Anti‐citrullinated protein antibodies are associated with neutrophil extracellular trap formation in rheumatoid arthritis

**DOI:** 10.1002/jcla.23662

**Published:** 2020-11-28

**Authors:** Siyu Wu, Wanchan Peng, Xianghui Liang, Wei Wang

**Affiliations:** ^1^ Department of Clinical Laboratory Xiangya Hospital Central South University Changsha China

**Keywords:** anti‐citrullinated protein antibodies, inflammation, neutrophil extracellular traps, rheumatoid arthritis

## Abstract

**Objectives:**

We evaluated the associations of anti‐citrullinated protein antibodies (ACPAs) and serological and cytological levels of neutrophil extracellular trap (NET) formation in rheumatoid arthritis (RA) patients.

**Methods:**

Serum levels of myeloperoxidase‐DNA and elastase‐DNA complexes (NET remnants) were examined in 51 patients with RA and 40 healthy controls using a modified enzyme‐linked immunosorbent assay. Neutrophils were isolated by density gradient centrifugation. IgG antibodies were purified by affinity chromatography. NET formation in RA and control neutrophils was assessed by microscopy in vitro. NETs were purified and co‐incubated with fibroblast‐like synoviocyte (FLS) cells. Interleukin (IL)‐6 and IL‐8 mRNA expression and protein levels in FLS cells were determined by real‐time polymerase chain reaction and enzyme‐linked immunosorbent assay, respectively.

**Results:**

In RA patients, NET remnants in the peripheral circulation were higher in extremely high ACPA titers when compared to in moderate ACPA titers. And IgG antibodies containing ACPA can stimulate neutrophils to form NETs in a concentration‐dependent manner. Furthermore, significantly higher expression of the pro‐inflammatory cytokines IL‐6 and IL‐8 is detected after FLS cells interacted with NETs which derived from neutrophils stimulated with ACPA‐containing IgG antibodies.

**Conclusions:**

Anti‐citrullinated protein antibodies may enhance NET formation and contribute to inflammation development in RA by stimulating NET formation, such as by subsequent activation of FLS cells by NETs.

## Graphical abstract

In rheumatoid arthritis (RA) patients, neutrophil extracellular trap (NET) remnants in the peripheral circulation were higher in extremely high anti‐citrullinated protein antibodies (ACPA) titers when compared to in moderate ACPA titers. And IgG antibodies containing ACPA can stimulate neutrophils to form NETs in a concentration‐dependent manner.

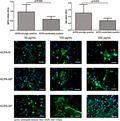

## 1 INTRODUCTION

Several cell types can form extracellular traps as a primary immune response when pathogens invade the body or other inflammatory stimuli. These cell types include neutrophils, monocytes, eosinophils, and mast cells. The mechanism by which neutrophil extracellular traps (NETs) are formed is known as NETosis. NETosis is a new feature, distinct from apoptosis and necrosis, first described by Brinkmann et al in 2004.^1^ Released NETs are characterized by extrusion of granular proteins, that is, myeloperoxidase (MPO), neutrophil elastase (NE), proteasomes, etc, bound to a meshwork of chromatin (DNA and its associated histone‐rich protein backbone) and other nuclear materials.^1^ The discovery of NETs expanded the known range of neutrophil defense mechanisms and prompted studies examining how neutrophils participate in immunological events in certain autoimmune diseases, including rheumatoid arthritis (RA).^2^, ^3^, ^4^, ^5^

Rheumatoid arthritis is a chronic autoimmune disease mainly involving inflammation of the cartilage and joints. A broad spectrum of autoantibodies is found in sera collected from patients with RA. However, anti‐citrullinated protein antibodies (ACPAs) and rheumatoid factor (RF) are the two autoantibodies most often used for the diagnosis and prognosis prediction of RA. ACPAs react with various deiminated proteins derived from a physiological process known as citrullination in RA. Although various autoantibodies are detected in the sera of patients with RA, ACPAs are of interest because they are highly specific for RA and play a vital role in its pathogenesis.^6^ Several proteins can be citrullinated and become potential autoantigens, including fibrinogen, keratin, α‐enolase, collagen, and histone.^7^, ^8^ Histone deimination is a crucial event in cell biology. Deiminated histones may regulate gene function and transcription, and histone deimination results from NET formation in neutrophils stimulated by infection or inflammatory stimuli.^9^ Recently, Pratesi et al^10^ suggested that ACPAs from patients with RA target citrullinated histone four contained in NETs. Thus, NETs may be a source of autoantigens in RA. Moreover, ACPA was suggested to stimulate neutrophils to form NETs, and NETs isolated from patients with RA may impact synoviocytes.^11^ Khandpur et al^11^ suggested a model for the roles of NETs in RA, in which NETs are associated with a series of events involved in disease pathogenesis, including protein deimination, autoantibody formation, fibroblast‐like synoviocyte (FLS) responses, and cytokine production. This model expands the range of known pathogenic mechanisms contributing to RA development. Researchers recently demonstrated interactions between NETs and FLS in vivo and in vitro, supporting that FLS‐neutrophil interactions promote pathogenic adaptive immunity in RA.^12^

However, the relationship between RA‐associated autoantibodies and NETosis needs further investigation, and the role of NETs derived from stimulation of autoantibodies in RA pathogenesis remains poorly understood. Here, we found that in RA patients, NET remnants in the peripheral circulation were higher in extremely high ACPA titers when compared to in moderate ACPA titers. Further, we examined the capacity of different concentrations of RA IgG antibodies containing ACPA to stimulate NET formation in neutrophils and investigated the effects of NETs induced by autoantibodies on FLS. Our results suggest a mechanism by which autoantibodies associated with NETs contribute to inflammation in RA.

## 2 MATERIALS AND METHODS

### 2.1 Human subjects

Peripheral blood (PB), serum, and synovial tissue were obtained from patients with RA at Xiangya Hospital. Enrolled patients fulfilled the 1987 American College of Rheumatology diagnostic criteria for RA^13^ and had no other systematic autoimmune diseases, infection, or major illness. Age‐ and gender‐matched healthy controls were enrolled by advertisement. The exclusion criteria for healthy volunteers were current or previous systematic autoimmune diseases, surgery, and current medication with corticosteroids or immunosuppressive agents. Several parameters associated with RA severity, namely 28‐joint disease activity score (DAS28), erythrocyte sedimentation rate (ESR), and serum C‐reactive protein (CRP) and ACPA were measured during a clinical visit. ESR was measured by the Westergren method. ACPA was detected using a commercial enzyme‐linked immunosorbent assay (ELISA) kit (Immunoscan CCPlus^®^; Euro‐Diagnostica) according to the manufacturer's recommendations. RF and CRP levels were determined using the immunonephelometric method. The demographic and clinical characteristics of 51 RA patients are presented in Table 1.

**TABLE 1:** Demographic data and disease indicators for the 51 rheumatoid arthritis patients included in the serologic tests

Characteristics	Descriptions
Sex (F/M)	36/15
Mean age (years)	53.1 ± 13.8
Disease duration (years)	6.8 ± 6.5
ESR (mm/h)	80.0 ± 32.9
CRP (mg/L)	8.1 (8.1–40.1)
ACPA	624.0 ± 574.5
ACPA‐N *n* (%)	10 (19.6)
ACPA‐MP *n* (%)	27 (52.9)
ACPA‐SP *n* (%)	14 (27.5)
DAS28	3.4 ± 0.9

Abbreviations: ACPA, anti‐citrullinated peptide antibodies; ACPA‐N, ACPA‐negative; ACPA‐MP, ACPA‐moderately positive; ACPA‐SP, ACPA‐strongly positive; DAS28, disease activity score in 28 joints.

We prepared three pools of sera from patients with RA and represented as an ACPA‐negative (ACPA‐N, ACPA < 25 U/ml) group, ACPA‐moderately positive (ACPA‐MP, 25 U/ml ≤ ACPA ≤ 1600 U/ml) group, and ACPA‐strongly positive (ACPA‐SP, ACPA > 1600 U/ml) group. Each group consisted of three patients. Sera were collected by centrifugation, and 500 μl of serum from each subject was used to make pools of sera. Synovial tissue was collected at the time of joint replacement surgery from a patient with RA. The clinical and laboratory characteristics of the patients provided serum samples and synovial tissues for this study were displayed in Table 2.

**TABLE 2:** The clinical and laboratory characteristics of the patients included in cytological tests

Specimen type	Patients no.	Age years	Sex	DAS28	ACPA U/ml	RF IU/ml	CRP mg/L	ESR mm/h	Purpose
Serum	RA1	46	M	3.86	<25	28	14.6	39	ACPA‐N serum pool
	RA2	38	F	4.55	<25	<20	31.8	116	
	RA3	43	F	4.67	<25	21	27.5	57	
Serum and whole blood	RA4	34	F	5.30	914	22	35.6	68	ACPA‐MP serum pool and neutrophil separation
	RA5	42	M	4.96	779	<20	45.1	28	
	RA6	37	F	4.80	876	<20	15.8	35	
Serum	RA7	48	F	4.89	>1600	25	18.9	49	ACPA‐SP serum pool
	RA8	43	F	5.29	>1600	<20	55.8	101	
	RA9	46	M	4.41	>1600	32	29.6	32	
PB	HC1	52	F	/	<25	<20	3.1	10	Neutrophil separation
	HC2	45	F	/	<25	<20	2.4	13	
	HC3	38	M	/	<25	<20	5.2	8	
ST	RA10	51	F	4.88	566	376	33.7	42	FLS separation and culture

Abbreviations: ACPA, anti‐citrullinated protein antibody; ACPA‐MP, ACPA‐moderately positive; ACPA‐N, ACPA‐negative; ACPA‐SP, ACPA‐strongly positive; CRP, C‐reactive protein; ESR, erythrocyte sedimentation rate; F, female; FLS, fibroblast‐like synoviocytes; HC, healthy control; M, male; PB, peripheral blood; RA, rheumatoid arthritis; RF, rheumatoid factor; ST, synovial tissue.

Informed, written consent was obtained from all subjects and the study was approved by the Ethics Committee of Xiangya Hospital, Central South University, where the study was performed.

### 2.2 NET remnant detection

Serum NET remnants were detected by an ELISA as described previously^14^ in 51 patients with RA and 40 age‐ and sex‐matched healthy controls. MPO‐DNA complexes and NE‐DNA complexes were evaluated. We used serum showing a weakly positive value (OD_405/490_ = 0.312) from one patient to serve as a positive control. Inter‐assay variability <20% was considered acceptable. The results were expressed as relative units (RUs), which were calculated by dividing the mean OD_405/490_ values of the samples by the mean OD_405/490_ value of the control sample.

### 2.3 IgG antibody purification

IgG antibodies were purified from the three pools of sera by affinity chromatography using a protein G Sepharose^®^ column (BioVision) according to the manufacturer's instructions. The purity of the purified antibody components was validated by sodium dodecyl sulfate polyacrylamide gel electrophoresis. An ACPA test was conducted to verify antibody activity.

### 2.4 Neutrophil isolation

Neutrophils were isolated by density gradient centrifugation using Polymorphprep^™^ solution (Axis‐Shield) according to the manufacturer's instructions. Cell viability was assessed by staining with trypan blue, and the purity of neutrophil samples was determined by flow cytometry (CD11b^+^Gr‐1^+^).

### 2.5 NETs detection

Neutrophil extracellular traps were detected using an immunofluorescent method as described previously with minor modifications.^11^ Briefly, 2 × 10^5^ of neutrophils were seeded onto 0.001% poly‐ʟ‐lysine (Sigma‐Aldrich)‐coated coverslips in tissue culture wells (2 ml/well) and allowed to settle for 1 h at 37°C in 5% CO_2_. The cells were then incubated with or without 10 ng/ml recombinant tumor necrosis factor (TNF)‐α (PeproTech) to determine the capacity for NET formation of neutrophils from patients with RA and healthy controls. Additionally, RA neutrophils were stimulated with 50, 100, and 200 μg/ml of ACPA‐N IgG, ACPA‐MP IgG, and ACPA‐SP IgG, respectively (each concentration in duplicate for each IgG antibody group). After adding TNF‐α or IgG antibodies, the cells were cultured for 48 h at 37°C in 5% CO_2_. The coverslips were washed with ice‐cold phosphate‐buffered saline (PBS) three times and then treated with 0.5% Triton X‐100^™^ in PBS for 2 min. The coverslips were washed again as previously described with ice‐cold PBS and then blocked with goat serum at 37°C for 30 min with 90% humidity. After washing, the cells were stained with mouse anti‐neutrophil elastase antibody (ab41179; Abcam; 1:200), followed by incubation with fluorescein isothiocyanate‐labeled goat anti‐mouse secondary antibodies (ab6785; Abcam; 1:400). After further washing steps, the nuclear materials were stained with ProLong^®^ Gold antifade reagent containing 4′,6‐diamidino‐2‐phenylindole (DAPI; Invitrogen; 1:2000). NETs were visualized using a fluorescent microscope (BX53; Olympus). NETs were defined as structures positive for both neutrophil elastase and DAPI. Each experiment was performed in triplicate, and two experienced observers who was familiar to relevant characteristics independently quantified the NETs. Ten fields of view were quantified for each coverslip. The results were expressed as the percentage area of the microscopic field of view occupied by the NETs.

### 2.6 NETs purification and quantification

NETs were purified as described previously.^11^ The purified NETs were quantified using Quant‐It^™^ PicoGreen^®^ dsDNA Reagent and Kits (Invitrogen) according to the manufacturer's instructions.

### 2.7 FLS separation and culture

Fibroblast‐like synoviocyte cells were separated from the synovial tissue of a patient with RA obtained during arthroplasty as described previously.^15^ The purity of the obtained FLS cells was evaluated by flow cytometry. Cells that were positive for vimentin staining and negative for CD68 staining were identified as FLS.

### 2.8 Interleukin (IL)‐6 and IL‐8 mRNA and protein detection

The concentration of FLS was adjusted to 1 × 10^6^ cells/ml, and 1 ml of the FLS suspension was added to each of six incubation wells (three test wells and three control wells). Test wells containing FLS were then incubated with purified NETs (1 μg/1000 cells) for 24 h at 37°C in 5% CO_2_, while control wells containing FLS were incubated without NETs. After incubation, the FLS cells were collected to determine the mRNA expression levels of IL‐6 and IL‐8 by real‐time PCR. The results were expressed as the mRNA expression levels of cytokines relative to that of β‐actin. The supernatants were collected to determine the protein concentrations of the cytokines. IL‐6 and IL‐8 concentrations in culture supernatants were determined with ELISA kits (R&D Systems) according to the manufacturer's recommendations.

### 2.9 Statistical analysis

Data were tested for normality using Kolmogorov‐Smirnov test in the total sample and within each group. If normally distributed, data were presented as the mean ± standard error, while not normally distributed, data were shown as medians (interquartile range). Differences between two groups were assessed by t test (normally distributed) or Mann‐Whitney *U* analysis (non‐normally distributed). The one‐way ANOVA test (normally distributed) and the Kruskal‐Wallis test (non‐normally distributed) were applied for comparison between three groups, and if there is statistical significance, further two‐to‐two comparison (the Newman‐Keuls test for normally distributed and the Kruskal‐Wallis test for non‐normally distributed) should be done. Statistical analysis was performed using SPSS 20.0 software (SPSS, Inc). A *p*‐value <0.05 was considered statistically significant.

## 3 RESULTS

### 3.1 Associations between serum NET remnants and ACPAs

**FIGURE 1:**
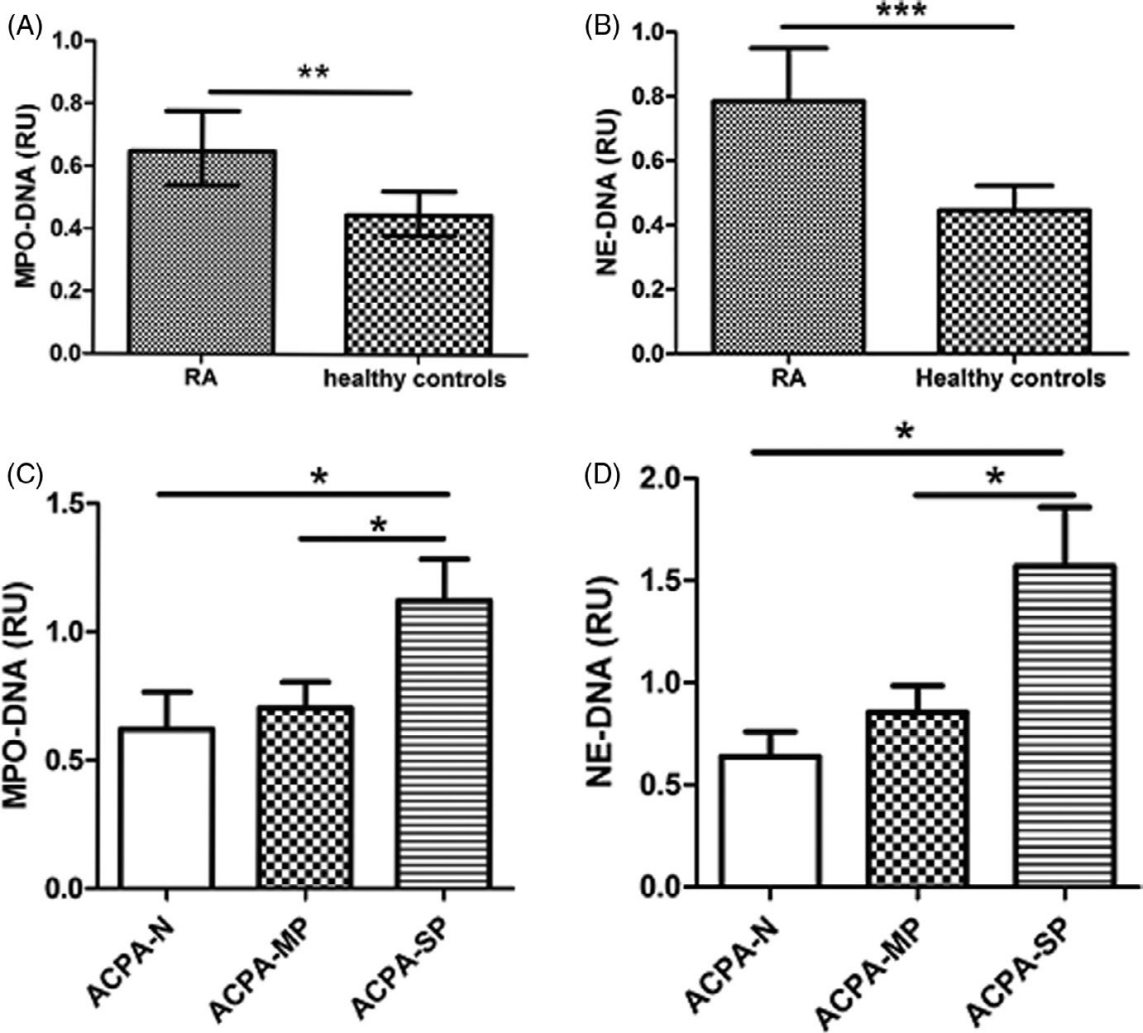
Neutrophil extracellular trap (NET) remnants were associated with titers of ACPAs in rheumatoid arthritis (RA) patients. Serum levels of myeloperoxidase (MPO)‐DNA and elastase (NE)‐DNA complexes (NET remnants) were examined in 51 patients with RA and 40 healthy controls using a modified enzyme‐linked immunosorbent assay. Patients with RA showed increased levels of NET remnants either defined as MPO‐DNA (A) or NE‐DNA (B) complexes. In 51 patients with different ACPA concentration, levels of MPO‐DNA complexes (C) and NE‐DNA complexes (D) were compared between ACPA‐N (<25 U/ml, *n* = 10), ACPA‐MP (25 U/ml ≤ ACPA ≤ 1600 U/ml, *n* = 27), and ACPA‐SP (>1600 U/ml, *n* = 14) group. The results were calculated by dividing the mean OD_405/490_ values of the samples by the mean OD_405/490_ value of the control sample (relative unit, RU). Data are presented as the medians (interquartile range). *p < 0.05; **p < 0.01; ***p < 0.001

The levels of NET remnants (MPO‐DNA complexes and NE‐DNA complexes) in RA patients and healthy controls are presented in Figure 1. Notably, RA patients exhibited significantly higher levels of MPO‐DNA complexes and NE‐DNA complexes than healthy controls (0.50 [0.40–1.05] vs 0.40 [0.30–0.60], and 0.70 [0.50–1.00] vs 0.60 [0.50–1.30], RU, median [interquartile range]; *p* = 0.009 and *p* < 0.001, Figure 1A,B). Furthermore, we compared the levels of NET remnants in RA patients with different ACPA concentration (ACPA‐N, ACPA < 25 U/ml; ACPA‐MP, 25 U/ml ≤ ACPA ≤ 1600 U/ml; ACPA‐SP, ACPA> 1600 U/ml). The results showed that the levels of MPO‐DNA complexes (*χ* ^2^ = 7.936, *p* = 0.019) and NE‐DNA complexes (*χ* ^2^ =10.107, *p* = 0.006) were different between the three groups. Further analysis revealed that the levels of MPO‐DNA complexes and NE‐DNA complexes were higher in RA patients with ACPA‐SP when compared to ACPA‐MP (1.05 [0.50–1.53] vs 0.50 [0.40–0.90] and 1.10 [0.78–2.75] vs 0.60 [0.40–0.90], RU, *p* = 0.048 and *p* = 0.022, Figure 1C,D), which were consistent with our previous results. Moreover, the levels of MPO‐DNA complexes and NE‐DNA complexes also showed increased in RA patients with ACPA‐SP, when compared to ACPA‐N (1.05 [0.50–1.53] vs 0.40 [0.38–0.98] and 1.10 [0.78–2.75] vs 0.50 [0.40–0.88], RU, *p* = 0.037 and *p* = 0.012, Figure 1C,D). No significance difference was found between RA patients with ACPA‐MP and ACPA‐N (*p* = 0.676 and *p* = 0.452).

### 3.2 RA‐derived neutrophils exhibited increased spontaneous NETosis and increased NETotic response to tumor necrosis factor (TNF)‐α

**FIGURE 2:**
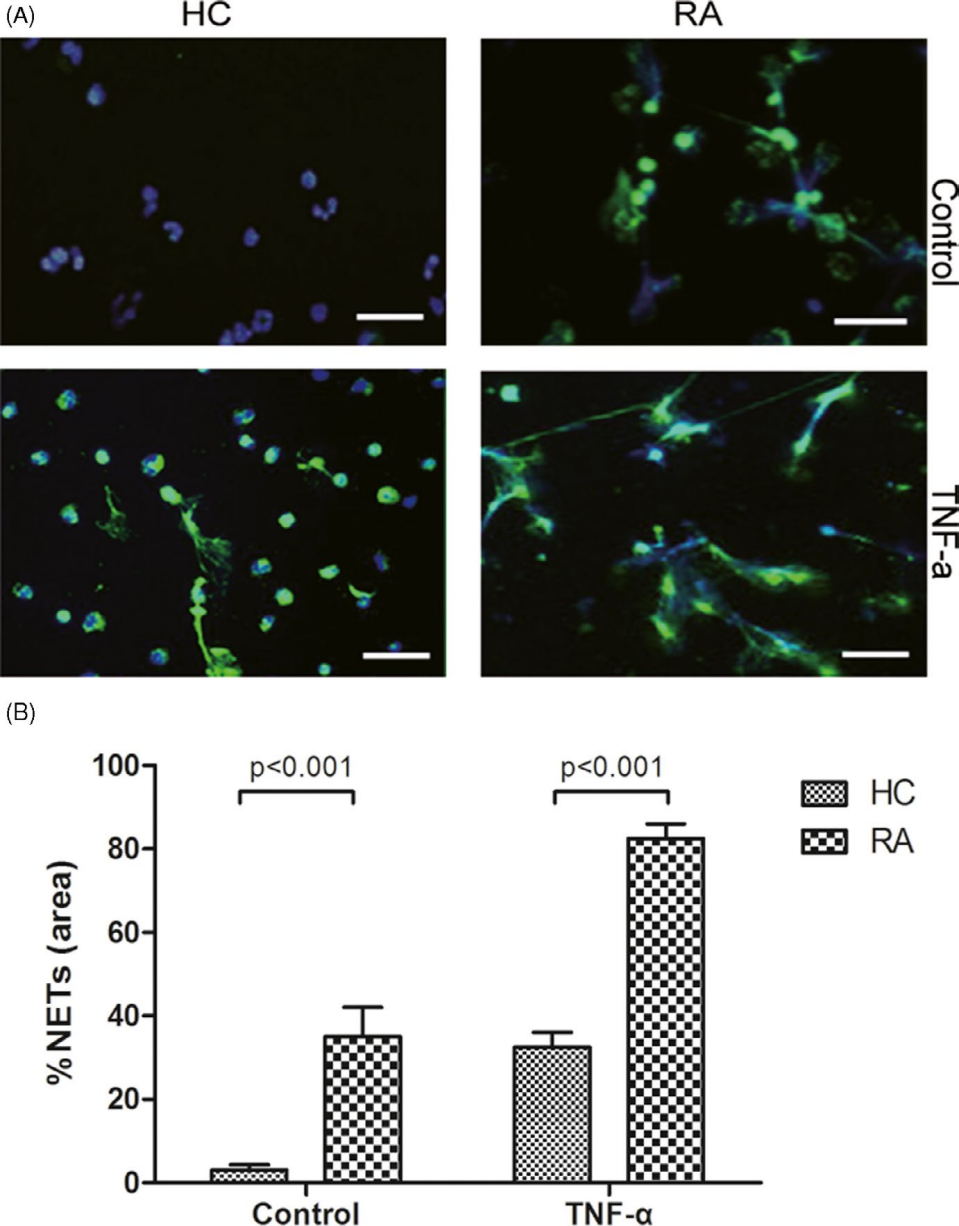
Rheumatoid arthritis (RA)‐derived neutrophils exhibited increased spontaneous NETosis and increased NETotic response to tumor necrosis factor (TNF)‐α. Neutrophils were isolated from patients with RA and healthy controls. Neutrophils from healthy controls and RA patients were stimulated with or without 10 ng/ml TNF‐α. NETs were detected in vitro by immunohistochemistry for NE (green) and 4′,6‐diamidino‐2‐phenylindole (DAPI; blue). Magnification, 20×; scale bar, 100 μm. Representative images demonstrating NETs formation are shown (A). Each experiment was performed in duplicate and two experienced observers quantified the NETs. At least 10 microscopic fields of view were quantified for each coverslip. The percentage area of NETs as a proportion of each microscopic field was recorded. The results were expressed as the mean ± SD (B)

Neutrophils were isolated from patients with RA and healthy controls, the viabilities of both cell populations were >90% tested by staining with trypan blue (data not shown), and purities were >95% according to the results from flow cytometry (Figure S1A). TNF‐α was added to determine the capacity for NET formation of neutrophils from patients with RA and healthy controls. Upon incubation without any stimulus, the area of NETs generated by the neutrophils from healthy controls was approximately 6.0% ± 2.5%, while neutrophils from RA patients were 15.1% ± 3.6% (Figure 2A,B *p* < 0.001). When exposed to TNF‐α, RA‐derived neutrophils were significantly more prone to NET formation compared to control cells (27.1% ± 9.7% vs 13.5% ± 2.9%, *p* < 0.001, Figure 2A,B).

### 3.3 NET formation was enhanced by ACPAs in a concentration‐dependent manner

In peripheral circulation, our data revealed that patients who had extremely high titers of ACPAs showed higher NET remnant levels. To further investigate whether ACPAs involved in NET formation, IgG antibodies were purified from ACPA‐N, ACPA‐MP, and ACPA‐SP RA patients. Results from SDS‐PAGE indicated that we obtained purified antibodies for the following experiments (Figure S1B). The concentration of ACPA in the purified IgG antibodies was <25 U/L for ACPA‐N, 898 U/L for ACPA‐MP, and >1600 U/L for ACPA‐SP, respectively (Figure S1C).

**FIGURE 3:**
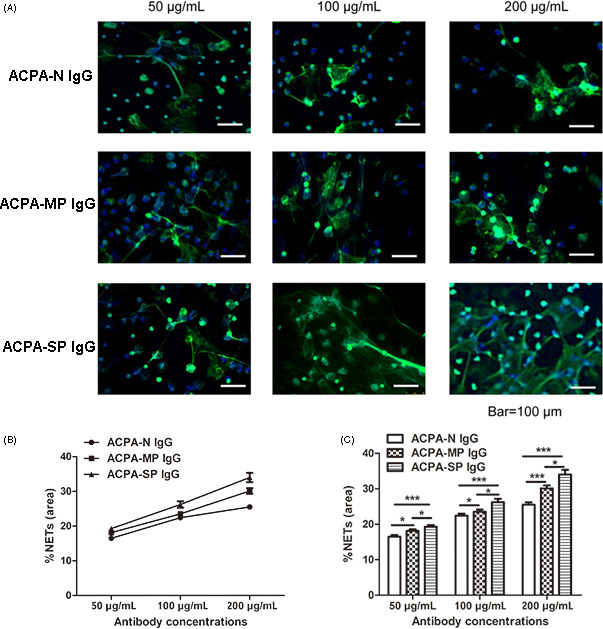
Neutrophil extracellular trap (NET) formation was enhanced by ACPA in a concentration‐dependent manner. Neutrophils isolated from patients with rheumatoid arthritis (RA) were stimulated with different concentrations (50, 100, and 200 μg/ml) of IgG antibodies purified from three pools of sera grouped according to their ACPA titers (ACPA‐N, ACPA‐MP, and ACPA‐SP groups). NETs were detected in vitro by immunohistochemistry for NE (green) and DAPI (blue). Magnification: 20×; scale bar, 100 μm. Representative images demonstrating NET formation are shown (A). NET formation was enhanced by ACPAs in a concentration‐dependent manner (B). Each experiment was performed in triplicate and two experienced observers quantified the NETs. At least 10 microscopic fields of view were quantified for each coverslip. The percentage area of NETs as a proportion of each microscopic field was recorded. The results were expressed as the mean ± SD. IgG antibodies containing higher titers of ACPAs induced more NET formation (C). *p < 0.05; **p < 0.01; ***p < 0.001. ACPA‐N, ACPA‐negative; ACPA‐MP, ACPA‐moderately positive; ACPA‐SP, ACPA‐strongly positive

Next, the neutrophils from RA patients were stimulated by purified IgG antibodies with different ACPA titers. The area of NETs increased as the IgG antibody concentration used for stimulation increased (Figure 3A). Compared to IgG lacking ACPAs (ACPA‐N IgG), IgG antibodies containing ACPAs (ACPA‐MP IgG and ACPA‐SP IgG) stimulated more NET formation. The amount of NET formation showed a positive tendency with the ACPA titer (Figure 3B). Results showed that the area of NETs generated by the neutrophils was different between the three groups (ACPA‐N IgG, ACPA‐MP IgG, and ACPA‐SP IgG group), when incubated with 50 μg/ml of IgG antibodies (*F* = 8.489, *p* < 0.001). Moreover, the differences were also exist between the three groups when incubated with 100 μg/ml (*F* = 7.143, *p* = 0.001) or 200 μg/ml (*F* = 18.97, *p* < 0.001) of IgG antibodies. Further analysis revealed that upon incubation with 50 μg/ml of IgG antibodies, the area of NETs was approximately 16.5% ± 3.6% for ACPA‐N IgG, 18.1% ± 3.8% for ACPA‐MP IgG (*p* = 0.020, ACPA‐N IgG vs ACPA‐MP IgG) and 19.3 ± 3.8% for ACPA‐SP IgG (*p* < 0.001, ACPA‐N IgG vs ACPA‐SP IgG; *p* = 0.046, ACPA‐MP IgG vs ACPA‐SP IgG). At 100 μg/ml, the areas of NET formation were 22.4% ± 4.3% for ACPA‐N IgG, 23.5 ± 5.1% for ACPA‐MP IgG (*p* = 0.048, ACPA‐N IgG vs ACPA‐MP IgG), and 26.2% ± 7.2% for ACPA‐SP IgG (*p* < 0.001, ACPA‐N IgG vs ACPA‐SP IgG; *p* = 0.020, ACPA‐MP IgG vs ACPA‐SP IgG). At 200 μg/ml, the areas of NET formation were 25.5% ± 5.1% for ACPA‐N IgG, 30.1% ± 6.3% for ACPA‐MP IgG (*p* < 0.001, ACPA‐N IgG vs ACPA‐MP IgG), and 34.0% ± 10.3% for ACPA‐SP IgG (*p* < 0.001, ACPA‐N IgG vs ACPA‐SP IgG; *p* = 0.014, ACPA‐MP IgG vs ACPA‐SP IgG; Figure 3C).

### 3.4 NETs up‐regulated IL‐6 and IL‐8 expression in FLS cells

**FIGURE 4:**
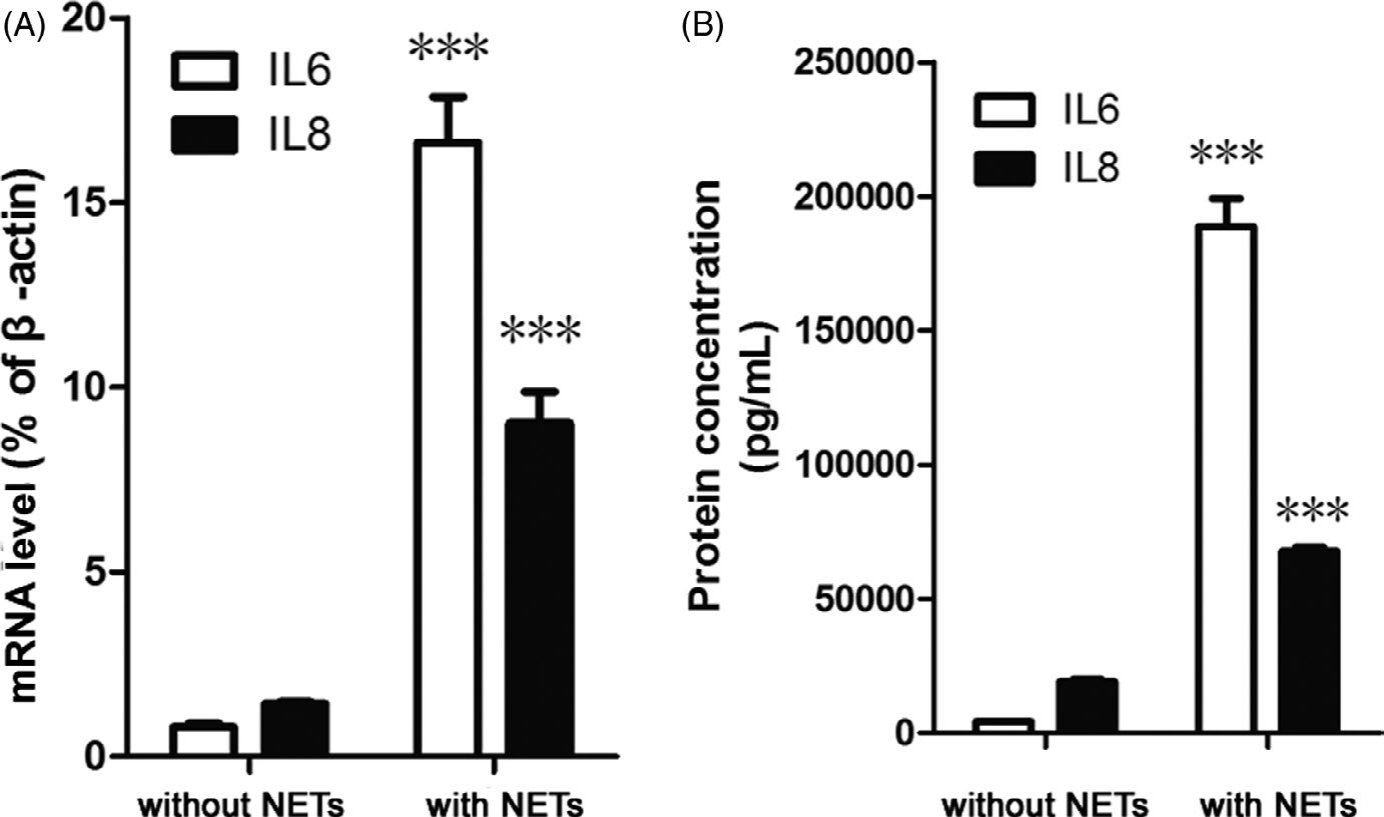
Neutrophil extracellular traps (NETs) up‐regulated the expression of IL‐6 and IL‐8 in FLS. FLS were incubated with or without purified NETs (1 μg/1000 cells) for 24 h at 37°C in 5% CO_2_. The expression of IL‐6 and IL‐8 was evaluated at the mRNA level by real‐time polymerase chain reaction or at the protein level by enzyme‐linked immunosorbent assay. The results were expressed as the relative mRNA expression relative to that of β‐actin (A) or calculated from a standard curve according to the recommendations of the manufacturer of the enzyme‐linked immunosorbent assay kit (B). Each experiment was performed in triplicate and the data were expressed as the mean ± SD. ***p < 0.001

Fibroblast‐like synoviocyte were separated from the synovial tissue of RA patients with a purity of approximately 90% (89.29%; Figure S2) and NETs were derived from neutrophils stimulated with ACPA‐containing IgG antibodies. FLS were incubated with or without purified NETs (1 μg/1000 cells) for 24 h. The expression of IL‐6 and IL‐8 was evaluated at the mRNA and protein level. Compared to cells without added NETs, the mRNA and protein levels of IL‐6 and IL‐8 were significantly increased in FLS co‐cultured with NETs (Figure 4A,B). In the NETs‐treated group, the mRNA levels of IL‐6 and IL‐8 were approximately 18‐ and 8.0‐fold higher than without‐NETs‐treated group (both *p* < 0.001, Figure 4A). Similarly, IL‐6 and IL‐8 protein concentrations in without‐NETs‐treated group culture supernatants were 4262 and 19,160 pg/ml, while in the NETs‐treated group, their concentrations were 188,727 and 67,773 pg/ml, respectively (Figure 4B, both *p* < 0.001).

## 4 DISCUSSION

In this study, we found that NET remnants in the peripheral circulation were associated with ACPA titers in RA patients. And IgG antibodies containing ACPA can stimulate neutrophils to form NETs in a concentration‐dependent manner. Moreover, NETs derived from IgG antibodies containing ACPA stimulation activate the FLS to express the pro‐inflammatory cytokines IL‐6 and IL‐8. These observations support that autoantibodies, like ACPAs, may participate in inflammation via NETs and expand the known range of mechanisms by which NETs contribute to RA pathogenesis.

In recent years, several research groups had focused on the involvement of NETs in the pathogenesis of autoimmune disorders, including RA, systemic lupus erythematosus, small‐vessel vasculitis, and antiphospholipid syndrome.^16^, ^17^, ^18^, ^19^, ^20^, ^21^ The presence of NETs had been demonstrated in synovial tissue, rheumatoid nodules, and skin from patients with RA.^11^ Additionally, in this paper, we showed that an increased concentration of NET remnants was detected in the circulation of patients with RA, which was consistent with our recent publications.^14^ These observations indicate that neutrophils in patients with RA are prone to NET formation.^18^ It is well‐known that neutrophils are commonly activated in RA by inflammatory stimuli, including cytokines. TNF‐α is likely an important stimulator of neutrophils within the joints of patients with RA to amplify the inflammatory response and contribute to tissue damage caused by neutrophils.^17^ In our study, we found that neutrophils isolated from patients with RA displayed increased NET formation compared to neutrophils from healthy controls in the absence of stimuli. Furthermore, neutrophils from patients with RA exhibited a greater increase in NET formation upon incubation with TNF‐α. Additionally, in the peripheral circulation, patients with RA displayed higher levels of NET remnants than healthy controls, further supporting the results of cytological experiments, which is consistent with our previous study.^14^

Anti‐citrullinated protein antibodies are the most important autoantibodies in the pathogenesis of RA and are also widely used for diagnosis and prognosis prediction. It was recently reported that exposure of neutrophils from patients with RA to ACPAs or IgM RF potently increased NET formation compared to that induced by control IgG.^11^ Another related study also mentioned that the serum nucleosome levels were higher in ACPA‐positive cases than in ACPA‐negative cases.^18^ Consistent with our previous study, data in this paper also revealed that in peripheral circulation, patients with ACPA‐SP showed higher NET remnant levels than patients with ACPA‐MP and ACPA‐N.^14^ Although the NET remnant levels slightly higher in patients with ACPA‐MP than ACPA‐N, no significance difference was found between ACPA‐MP and ACPA‐N. This may due to the limited sample number. To further investigate the association between ACPAs and NET formation in RA, we combined IgGs into three pools representing patients who had undetectable, moderate, and high ACPA titers. Each pool of IgGs was used at three concentrations (50, 100, and 200 μg/ml) to stimulate neutrophils isolated from patients with RA. We observed that ACPAs enhanced NET formation by neutrophils in a concentration‐dependent manner. Autoantibodies can activate cytokine‐primed neutrophils, which was systematically evaluated in ANCA‐associated vasculitides.^22^ The exact signaling pathway by which autoantibodies activate NET formation by neutrophils is unclear. A possible mechanism is that autoantibodies or its immune complexes bind to neutrophils via Fcγ receptors, which subsequently initiate cellular responses such as chemotaxis, phagocytosis, bacterial killing, and reactive oxygen species generation catalyzed by NADPH oxidase.^23^ The generation of ROS via NADPH oxidase is an important early step in NET formation by neutrophils. This viewpoint was also supported in a study conducted by Chowdhury et al.^18^ It should be emphasized that large quantities of cytokines such as IL‐1β, IL‐6, TNF‐α, transforming growth factor‐β and IL‐8 are detected in the synovial fluid and peripheral circulation of patients with RA.^24^, ^25^ It has been indicated that neutrophils in patients with RA are persistently stimulated by these cytokines and adopt a primed status, in which they express abundant Fcγ receptors compared to resting cells.^26^, ^27^, ^28^ In the present study, neutrophils incubated with IgG antibodies containing a high titer of ACPAs showed increased NET formation, suggesting that the cell surface hosts a large quantity of Fcγ receptors for ACPA binding. Presumably, ACPAs may be prone to binding primed neutrophils via Fcγ receptors to initiate cellular responses leading to NET generation.

Neutrophil extracellular traps can not only serve as a source of autoantigens for ACPA production, but also act as a stimulus to activate FLS to secrete pro‐inflammatory cytokines. Notably, different stimuli may induce distinct protein cargos in NETs.^11^ In our study, NETs purified from incubation of neutrophils with IgG antibodies containing ACPA up‐regulate the expression of IL‐6 and IL‐8 in FLS from RA patients. One mechanism by which FLS cells contribute to bone erosion is mediated via the synthesis and secretion of mediators of inflammation, including IL‐6 and IL‐8.^29^ Previous studies indicated that IL‐6 and IL‐8 expression in FLS are regulated by several transcription factors including nuclear factor‐κB, C/EBPβ, and c‐Jun.^30^, ^31^ However, the molecular components of NETs that activate FLS and signaling pathways and transcription factors contributing to up‐regulation of IL‐6 and IL‐8 require further analysis.

Several cellular events are thought to be critical for regulating NET formation by neutrophils; these events include the production of reactive oxygens species, migration of neutrophil elastase and later myeloperoxidase to the nucleus, and histone modification and chromatin decondensation. Citrullinated histones are a potential source of autoantigens for ACPA production in patients with RA. Additionally, ACPAs may further stimulate neutrophils to form more NETs, subsequently activating FLS cells to synthesize and secrete IL‐6 and IL‐8. Presumably, these associated biological events participate in the perpetuation and amplification of anatomically localized inflammation in RA.

Despite the clinically relevant findings in this study, there are some limitations. The sample numbers in the three group (ACPA‐N, ACPA‐MP, and ACPA‐SP group) are limited. And the precise mechanism by which ACPAs stimulate NETs remains unknown. Thus, further studies are needed to confirm our conclusion and to investigate the precise mechanisms by which ACPAs stimulate NETs and the mechanism that ACPAs together with NETs involved in RA pathogenesis.

## CONFLICT OF INTEREST

None.

## Supporting information

Supplementary Material

## Data Availability

The data that support the findings of this study are available from the corresponding author upon reasonable request.
