# Supplementary material for: Anti‐citrullinated protein antibodies are associated with neutrophil extracellular trap formation in rheumatoid arthritis
Source: J Clin Lab Anal. 2020 Nov 28;35(3):e23662. doi: 10.1002/jcla.23662 (PMC7957993; doi:10.1002/jcla.23662)
Supplement: Supplementary file 1 — Supplementary Material [file JCLA-35-e23662-s001.doc]

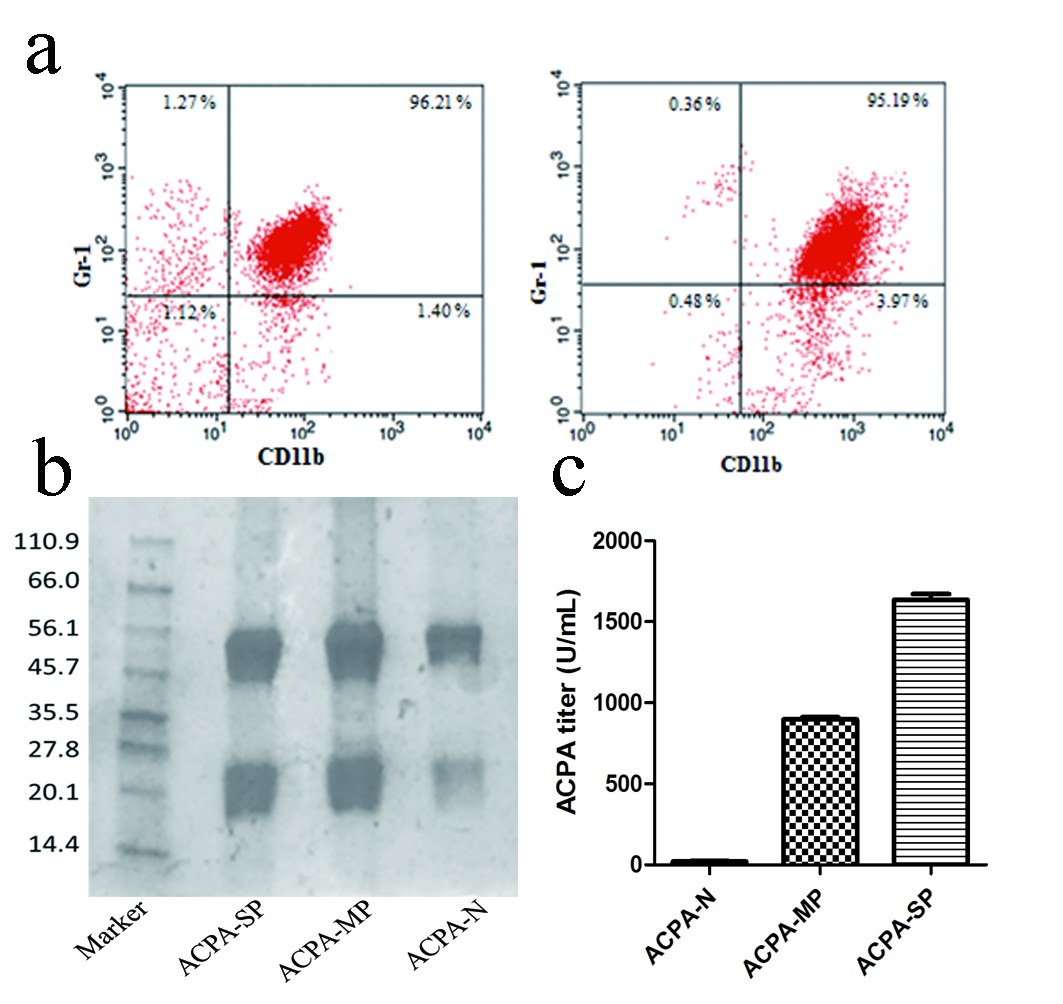


**Fig. S1** Isolation and purification of neutrophils and antibodies. Neutrophils were isolated from patients with RA and healthy controls. The purities was determined by flow cytometry (a). IgG antibodies were purified from three pooled serum samples from patients with RA representing ACPA-negative (ACPA-N), ACPA-moderately positive (ACPA-MP), and ACPA-strongly positive (ACPA-SP). The purity of the purified antibody components was validated by SDS-PAGE (b). The concentrations of ACPA in the three pooled serum samples were detected using a ELISA kit.


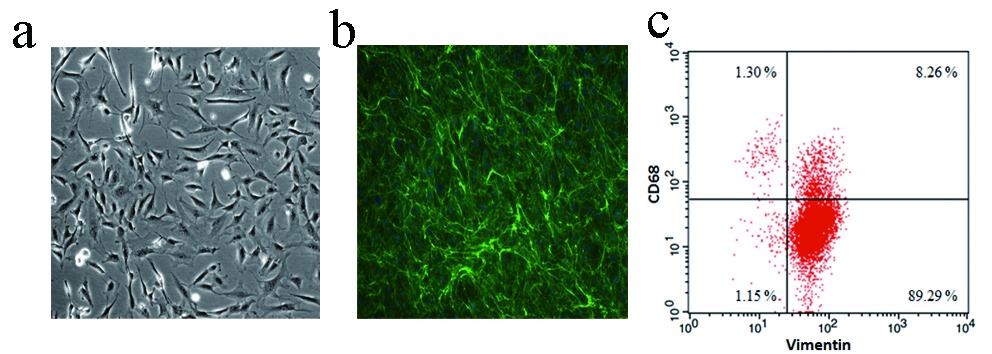


**Fig. S2** Separation of fibroblast-like synoviocytes (FLS) from synovial tissue. FLS were separated from the synovial tissue of a patient with RA obtained at arthroplasty. The cells were visualized using an ordinary optical microscope (a) or a fluorescence microscope with vimentin and DAPI staining (b). Magnification: 20×; scale bar, 200 μm. The purity was evaluated by flow cytometry, and cells that were positive for vimentin staining and negative for CD68 staining were identified as FLS (c).
